# The Impact of Epidemiological Trends and Guideline Adherence on Candidemia-Associated Mortality: A 14-Year Study in Northeastern Italy

**DOI:** 10.3390/jof11050400

**Published:** 2025-05-21

**Authors:** Fabiana Dellai, Alberto Pagotto, Francesco Sbrana, Andrea Ripoli, Giacomo Danieli, Alberto Colombo, Denise D’Elia, Monica Geminiani, Simone Giuliano, Assunta Sartor, Carlo Tascini

**Affiliations:** 1 Department of Medicine (DMED), University of Udine, 33100 Udine, Italy; 2 Infectious Diseases Division, Azienda Sanitaria Universitaria Friuli Centrale (ASUFC), 33100 Udine, Italy; alberto.pagotto@asufc.sanita.fvg.it (A.P.);; 3 Lipoapheresis Unit and Reference Center for Inherited Dyslipidemias, Fondazione Toscana Gabriele Monasterio, 56124 Pisa, Italy; 4 Independent Researcher, 36100 Vicenza, Italy; 5 Microbiology Unit, Azienda Sanitaria Universitaria Friuli Centrale (ASUFC), 33100 Udine, Italy

**Keywords:** *Candida* bloodstream infections, EQUAL *Candida* score, epidemiology, guideline adherence, surveillance programs

## Abstract

Invasive candidiasis represents a major global health concern, with incidence and mortality rates expected to rise due to medical advancements and unavoidable risk factors. This retrospective, multicentric study was conducted in eight hospitals in a northeastern Italian region, enrolling adult patients diagnosed with candidemia from 1 January 2018 to 31 December 2022. Epidemiological trends and clinical characteristics were analyzed and compared to those from a prior regional study (2009–2011), allowing a fourteen-year comparative evaluation. A shift in species distribution was observed, with a decline in *Candida albicans* (from 65.7% to 57.8%) and a rise in non-*albicans* species, particularly the *Candida parapsilosis* complex (from 16.1% to 18.2%). Guideline adherence was assessed applying the EQUAL *Candida* score; scores ≥ than 11.5 were independently associated with improved in-hospital survival (HR 3.51, *p* < 0.001). Among individual score components, empiric echinocandin therapy and central venous catheter removal correlated with better outcomes. Centers with routine infectious disease (ID) consultations showed higher survival and adherence, reinforcing the value of specialist involvement. These findings support local epidemiological and management practice surveillance program adoption to address context-specific gaps, promote the adoption of best practices in *Candida* BSI management—as expanded ID specialist consultations and education programs—and, ultimately, reduce candidemia-related mortality rates.

## 1. Introduction

Despite the advances in diagnostics and antifungal therapies, invasive candidiasis (IC) remains a formidable global health challenge marked by persistently high incidence and mortality rates, along with an economic burden of approximately $1.8 billion in the United States alone [1].

A recent review estimated that approximately 1.57 million cases of IC occur annually worldwide, with candidemia accounting for about half of them, leading to an estimated incidence rate of 3 to 8 cases per 100,000 people in the general population [2]. In the United States, the 36% of all-cause in-hospital mortality is associated with candidemia, while in Europe, the 90-day mortality rate approaches 43%, with an attributable mortality rate estimated to be between 22% and 27% [3,4,5,6,7,8,9].

According to recent data, candidemia mortality rates in Italy range from 28% to 41.6% [10,11,12,13,14,15,16], with an estimated annual number of cases between 2455 and 13,554 [17]. The cost of managing a single bloodstream *Candida* infection is estimated to range from EUR 8303 to EUR 8401, resulting in an overall estimated annual economic burden on the Italian National Health Service that exceeds EUR 80 million, considering exclusively the costs related to diagnosis, treatment, and management [17].

IC can be partly viewed as a disease intrinsically tied to healthcare innovations, and is largely driven by its strong association with several unavoidable risk factors. Improved survival among frail, multimorbid patients, alongside increasingly complex surgical procedures, novel chemotherapy, and immunosuppressive regimens, and the widespread use of endovascular devices in outpatient settings, has collectively expanded the pool of vulnerable individuals at risk from invasive candidiasis [4]. However, the stagnation in survival improvements cannot be attributed solely to advancements in knowledge. The shift toward a higher prevalence of non-*albicans Candida* (NAC) species characterized by a reduced susceptibility pattern is occurring at an alarming rate, stimulated by climate changes and selective pressure from antifungal misuse in both medical and agricultural settings. The selection of multidrug-resistant (MDR) and extensively drug-resistant (XDR) *Candida* species is increasing worldwide, particularly in *Nakaseomyces glabratus* (formerly *C. glabrata*), where co-resistance to azoles and echinocandins is becoming more common [18,19,20]. This phenomenon, paired with the recent emergence of *Candida auris*—a new *Candida* species characterized by high transmissibility, persistence in healthcare environments, and frequent resistance to multiple classes of antifungal agents—represent a severe incipient threat to public health globally [21].

Another directly addressable key factor contributing to the high IC burden is suboptimal adherence to clinical guidelines. Despite the fact that guidelines for diagnosing and managing IC have not been updated since 2016, and the introduction of the user-friendly EQUAL *Candida* score in 2018, multiple studies highlight low adherence to clinical guideline recommendations globally, with non-compliance being associated with increased mortality risk [7,22,23,24].

## 2. Materials and Methods

This retrospective, multicentric, observational study enrolled all adult patients (≥18 years) who were hospitalized in eight public hospitals in a northeastern region of Italy (Friuli) and who developed candidemia between 1 January 2018, and 31 December 2022. Among the hospitals included, seven were peripheral secondary care hospitals and will be collectively referred to as “spoke hospitals” (SH), whereas only one of the hospitals included in the study served as a tertiary university-affiliated hospital (hub hospital—HH). Candidemia was defined according to the European Society of Clinical Microbiology and Infectious Diseases (ESCMID) criteria [25,26]. For each patient, only the first episode of candidemia was recorded and cases of mixed candidemia were excluded. The time of collection of the first positive blood culture was designated as the index time for all time-dependent variables and outcomes related to *Candida* bloodstream infections (BSIs).

The primary outcome of this study was to define the epidemiology, management patterns, and clinical and microbiological outcomes of candidemia across the Friuli subregion. Crude mortality was calculated during hospitalization and at 30 and 90 days after candidemia diagnosis.

Adherence to current clinical guidelines (Infectious Diseases Society of America—IDSA—and ESCMID) was assessed using the EQUAL *Candida* score, an easy to apply flowchart which quantifies and aggregates the most robust recommendations in optimal management of candidemia [27].

### 2.1. Microbiological Analysis

*Candida* spp was isolated from blood samples using the BD BACTEC^TM^ FX system (Becton, Dickinson, Inc., Sparks, MD, USA). Species identification was performed using both conventional and Matrix-Assisted Laser Desorption/Ionization Time-of-Flight mass spectrometry technology (MALDI–TOF system). *C. parapsilosis* strains were identified only at complex level. Susceptibility to amphotericin B, echinocandins (micafungin and anidulafungin), and azoles (fluconazole, itraconazole, posaconazole, and voriconazole) was detected using broth microdilution dedicated panels MICRONAUT–AM (Bruker Daltonics GmbH and Co. KG, Bremen, Germany). MIC values were interpreted according to species-specific clinical breakpoints as established by European Committee on Antimicrobial Susceptibility Testing (EUCAST), updated to the last version available [28,29]. No changes in the microbiological laboratory techniques at our hospital were undertaken during the study period.

### 2.2. Statistical Analysis

Clinical and microbiological data were retrieved from electronic and paper health records, collected anonymously in a standardized database and de-identified before statistical analysis. Continuous variables were compared using the Wilcoxon rank sum test, while categorical variables were analyzed with Pearson’s Chi-squared test. The false discovery rate (FDR) correction was applied when managing multiple comparisons. Survival analyses were performed using the survival and survminer packages in R, focusing on Kaplan–Meier curves and log-rank tests. Cox regression models were applied in identified optimal mortality-predictive cut-off score in adherence sub-analysis. Odds ratios (ORs) were estimated using logistic regression models.

All statistical analyses were conducted in R version 4.4.2 (31 October 2024)—“Pile of Leaves” using Rstudio 2024.12.0. A two-tailed *p*-value < 0.05 was considered statistically significant [30].

## 3. Results

### 3.1. Epidemiology, Risk Factors, and Clinical and Microbiological Outcomes

#### 3.1.1. Epidemiology and Clinical Outcomes

Among the 384 *Candida* spp. isolated from blood culture during the study period, 341 fulfilled the inclusion criteria and were enrolled in the study. Full details of patients’ demographic and clinical characteristics complete the description and can be found in the Supplementary Material [Table S1]. The majority of patients were male (N = 199, 58%), and more than half of candidemia episodes (N = 230, 67.4%) occurred in patients older than 70 years. At the time of *Candida* BSI diagnosis, over 90% of patients (N = 322) had at least one comorbidity. Specifically, 29% had a solid organ malignancy, 18% were diabetic, 6.5% had liver disease, and 5.6% presented with a hematological malignancy. Among the risk factors for candidemia, almost 69% of patients (N = 234) presented with an endovascular device—central venous catheter (CVC), peripherally inserted central catheter (PICC), or midline—placed before diagnosis. Additionally, 229 patients (67.2%) received total parenteral nutrition (TPN), and in 317 cases (92.9%) at least one antibiotic was administered for ≥7 days within the 30 days preceding candidemia onset. The most commonly used antibiotics included penicillins (76%), carbapenems (41%), and fluoroquinolones (13%). Concomitant bacteremia was detected in 20.2% of patients (N = 69), with Gram-positive and Gram-negative bacteria accounting for 63.8% and 23.2% of all the infections, respectively; the remaining cases involved mixed bacterial flora. One hundred and forty-two patients (N = 142; 39.8%) had undergone surgery in the two months prior to candidemia diagnosis and approximately half of them (N = 69, representing the 20% of the total population—69/341) underwent an abdominal procedure. Ninety-eight patients (N = 98; 29%) were admitted to the intensive unit care (ICU) during the hospitalization. The overall in-hospital mortality rate was 48.4% (N = 165/341) and increased to 51% (N = 174/341) within the first 30 days after candidemia diagnosis. This percentage had further increased to 60.4% (N = 206/341) at the 90-day follow-up.

#### 3.1.2. Protective Factors

Univariate logistic regression was performed to evaluate potential protective factors among candidemic patients diagnosed between 2018 and 2022 (N = 341). The outcome variable was defined as 30-day survival; therefore, variables with an odds ratio (OR) > 1 were considered protective factors, while those with an OR < 1 were considered risk factors. As expected, mortality rates were significantly higher among elderly patients and subjects with a higher Charlson Comorbidity Index score (CCI). Aging (non-survivors’ median age: 78 years [SD: 70–84] vs. survivors’ median age: 74 years [SD: 63–82], OR = 0.95), multimorbidity (non-survivors’ median CCI score: 6 [SD: 5–9] vs. survivors’ median CCI score: 5 [SD: 3–7], OR = 0.82), metastatic solid tumors (OR = 0.49), hospitalization in internal medicine wards (IMWs) (OR = 0.36), and failure in endovascular device removal (OR = 0.31) were significantly associated with poorer outcomes. Conversely, admission to the hub hospital (OR = 2.08), hospitalization in surgical units (OR = 3.03), *C. parapsilosis* complex-related infections (OR = 2.49), ophthalmologic examination (OR = 12.48), and echocardiography (OR = 6.79) emerged collectively as protective factors [Table 1].

#### 3.1.3. Candida BSI Management

Of the 294 patients (86.2%) who received antifungal therapy, echinocandins were the first-line treatment in 171 patients (58.2%), with caspofungin prescribed in around three-quarters of these cases (N = 131, 76.6%). Nearly all the remaining patients (N = 123) received fluconazole as a first-line treatment (N = 122, 99.2%). Liposomal amphotericin B (L–AmB) was only sporadically used (N = 2, 0.68%), hence, analyses for this drug were not performed to avoid important interpretative bias. Ophthalmological evaluation (N = 119, 35%) and echocardiography (N = 111, 32.6%) were executed for approximately one-third of the enrolled patients, resulting suggestive for ocular and cardiac infective involvement in 5 (4.1%) and 3 (2.7%) patients, respectively.

#### 3.1.4. Microbiological Outcomes

Follow-up blood cultures were performed in 215 patients (63%), with a median duration of bloodstream invasion of 6 days (interquartile range—IQR: 4–10 days). A significantly shorter infection clearance time was observed in patients who had endovascular devices removed within the first 72 h after diagnosis (median candidemia duration: 5 days [IQR 3–10] vs. 7 days [IQR 6–13], *p* = 0.006). Similarly, patients treated with echinocandins or L–AmB achieved early hemoculture sterilization (5 days [IQR 4–8] vs. 9 days [IQR 7–14], *p* < 0.001). Differently from patients with ocular involvement, only patients diagnosed with *Candida* endocarditis exhibited a significantly prolonged time of BSI eradication (median candidemia duration: 7 days [IQR 5–12] vs. 10 days [IQR 2–20], *p* = 0.01).

### 3.2. Adherence to Clinical Guideline Recommendations

To minimize the potential bias related to early mortality and ensure a valid comparison with similar studies, only patients who survived beyond seven days after candidemia diagnosis were included (N = 275). Table 2 presents the overall adherence levels.

When assessing the overall performance of the eight hospitals included in the study, strict compliance was observed only for microbiological practices. Conversely, adherence to antifungal treatment recommendations and diagnostic follow-up procedures remained suboptimal, with marked differences between hub (HH) and spoke hospitals (SH).

Specifically, administration of echinocandin as first-line therapy was achieved in only 61% of cases (HH: 79% vs. SH: 38%). Similarly, follow-up blood cultures (HH: 90% vs. SH: 61%), appropriate treatment duration (HH: 86% vs. SH: 45%), and echocardiography (HH: 56% vs. SH: 28%) were significantly more frequently performed in the hub hospital. In our cohort, a shift to azole-based oral therapy was performed in only 29% of the patients, with significantly higher rates occurring in the hub hospital (HH: 32% vs. SH: 21%).

## 4. Discussion

### 4.1. Epidemiology, Risk Factors, and Clinical and Microbiological Outcomes

Candidemia remains a major cause of morbidity and mortality worldwide, and is characterized by an increasing prevalence and shifting epidemiology.

To better understand local long-term trends, we compared current data with results retrieved by a similar study conducted in the same hub hospital from 2009 to 2011, enabling a fourteen-year comparison analysis [34].

**Figure 1:**
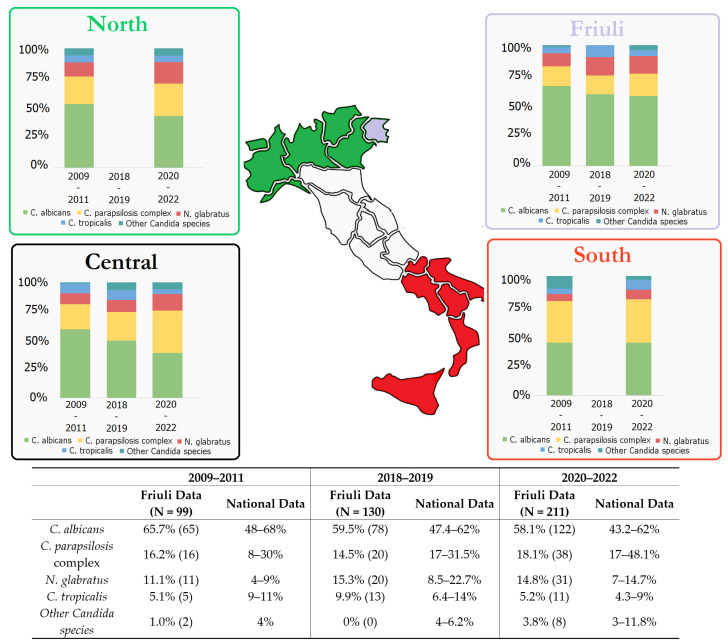
Temporal changes in *Candida* species distribution at a national and regional level. Data were stratified by geographic region (based on latitude) to highlight local trends. It should be noted that the percentage values represent mean estimates, and significant discrepancies may exist between neighboring regions. In the Friuli dataset, the “Other Candida species” category includes *Candida dubliniensis*, *Candida famata*, *Clavispora lusitaniae* (formerly *Candida lusitaniae*), *Kluyveromyces marxianus* (formerly *Candida kefyr*), and *Pichia kudriavzevii*. In contrast, the corresponding group in the national dataset also includes *Candida auris*. Italian data were extrapolated from refs. [10,11,12,13,14,15,37,38,39].

Similar to global trends, we observed a decline in *C. albicans* BSIs from 65.7% (2009–2011) to 58.1% (2020–2022), alongside a progressive increase in non-*albicans Candida* (NAC) species [Figure 1] [35,36].

However, as showed in Figure 1, our regional epidemiology showed unique features compared to national data [10,11,12,13,14,15,37,38,39,40]. While *C. parapsilosis* complex was the second most common species, its incidence was lower than the national median (18.1% vs. 24.5%). *N. glabratus* prevalence remained stable (~15%), suggesting convergence with national trends in the near future. No *C. auris* outbreaks were identified, but from 2020 onward, rare NAC species (including *Candida dubliniensis, Candida famata, Clavispora lusitaniae* (formerly *Candida lusitaniae),* and *Kluyveromyces marxianus* (formerly *Candida kefyr)*) began to emerge, comprising 2.6% of isolates. Though limited, this shift may reflect evolving local ecology and warrants continued monitoring.

Compared to national and European data [14,40], our antifungal susceptibility patterns appear more favorable, as *C. albicans* remained fully azole-susceptible, and *C. parapsilosis* complex maintained high susceptibility to fluconazole (92.1%) and echinocandins (>94%). However, early signs of reduced susceptibility to anidulafungin (5.2%) and micafungin (2.7%) were noted. While *N. glabratus* showed decreased fluconazole susceptibility in 6.5% of isolates, no echinocandin resistance was detected. Full susceptibility data are provided in the Supplementary data [Tables S2 and S3].

After grouping the four rarest species together (*C. dubliniensis, C. famata, C. lusitaniae,* and *K. marxianus*) a statistically significant effect on mortality was observed in each follow-up point (intrahospital mortality: χ^2^_4_ = 11.9, *p* = 0.018; 30-day mortality: χ^2^_4_ = 14.6, *p* = 0.006, and 90-day mortality: χ^2^_4_ = 11.0, *p* = 0.027). Consistent with SENTRY and ECMM data, mortality rates were the highest among patients with *N. glabratus* BSIs, likely due to its tropism for elderly, oncologic, and solid organ transplant (SOT) recipients [19,41]. This result is particularly worrisome considering the incipient threat represented by the emergence of azole- and echinocandin-resistant strains that significantly limits antifungal therapeutic strategies [19,41]. Since, in our setting, no MDR or XDR isolates were found, our poorer outcome may be potentially attributed to the extensive use of standard-dose fluconazole as a first-line empirical antifungal treatment in peripheral hospitals. In contrast, *C. parapsilosis* complex infections showed the lowest mortality, likely due to lower virulence and a good susceptibility profile.

As here confirmed, epidemiological data extrapolated from national and supranational levels cannot always be systematically applied locally. Region-specific surveillance programs are essential to monitor shifts in local epidemiology, detect early potential outbreaks, and guide tailored antifungal treatment strategies’ implementation.

Focusing on changes in the prevalence of risk factors over the ten-year period, a significant increase in antibiotic use in the 30 days prior to candidemia diagnosis was observed (from 83.8% to 91%, *p* = 0.006), while *Candida* colonization rates significantly dropped (from 58.6% to 10.5%, *p* < 0.001), likely influenced by a reduction in this screening practice performance. Also, a three-fold higher frequence in endovascular catheter use has been documented in recent years (from 24.2% to 69.3%).

Both in-hospital and 30-day mortality rates have remained consistently high, at approximately 50%, throughout the fourteen-year analysis period [Table 3]. This occurred alongside a substantial shift toward an older affected population, with the median age at candidemia diagnosis increasing from 63 years (2009–2011) to 73 years (2018–2022). In comparison to other European cohorts, our study reported higher mortality, particularly at 30 days (50% vs. 43%), likely reflecting the older median age of our population (73 vs. 65–68 years) [2,5,7,22,34,35,42]. This association is supported by prior evidence linking being aged over 70 years with significantly poorer outcomes [16,43,44].

The aging trend in our cohort was accompanied by a significant rise in CCI scores, despite declining rates of several comorbidities—including cardiovascular disease (from 57.6% to 39.5%, *p* < 0.001), metastatic malignancy (from 25.3% to 11%, *p* = 0.005), hematological malignancies (from 8.1% to 7.1%, *p* = 0.047), chronic hepatopathy (from 20.2% to 5.7%, *p* < 0.01), and chronic steroid use (from 21.2% to 2.9%, *p* < 0.001)—demonstrating a widespread diffusion of IC outside the recognize high-risk populations [11,43,45,46,47,48].

A CCI score of at least four is widely recognized as an independent predictor of increased mortality in patients with candidemia [24,34,49,50]. In our cohort, the median CCI was five, indicating a higher baseline burden of comorbidities, which may partially account for the elevated mortality rates observed here [Table 3].

### 4.2. Adherence to Clinical Guideline Recommendations

Effective management of candidemia, through strict adherence to clinical guidelines, is vital for achieving favorable patient outcomes. Both the international guidelines from the IDSA [51] and the ESCMID [25,26] recommend initiating early empiric therapy with an echinocandin, prompt CVC removal, and follow-up blood cultures every 48 h until clearance, as well as echocardiographic and ophthalmologic evaluations for all patients receiving *Candida* BSI diagnosis. In case of primary candidemia, antifungal therapy should be continued for 14 days following the first negative blood culture; while in case of septic embolism or abscess development, treatment duration should be tailored based on the site of embolization, presence of synthetic prosthetic material, and the clinical and microbiological evolution of the infection [25,26,51]. A key distinction between American and European guidelines lies in the use of azoles, which are permitted by IDSA in selected non-neutropenic, hemodynamically stable patients without recent azole exposure or risk factors for *N. glabratus* or *P. kudriavzevii* [51].

When comparing our study results with the III ECMM *Candida* study data (EU) [22], and hospitals in the Friuli region (HFR) demonstrated higher adherence to microbiological guidelines (HFR: 100% vs. EU: 44–46%), blood culture follow-up (HFR: 70% vs. EU: 35%), empiric echinocandin initiation (HFR: 61% vs. EU: 42%), and appropriate treatment duration (HFR: 84% vs. EU: 21%). In contrast, lower compliance was observed in CVC removal within 72 h of diagnosis (HFR: 16% vs. EU: 40%) [Table 2]. It is important to note that the data from the pan-European study reflect diverse healthcare settings and policies.

Compared to institutions located in high-income countries (e.g., North America and Germany), HFR showed lower adherence to recommended practices regarding echinocandin initiation, CVC removal, and follow-up blood cultures, confirming how the management of *Candida* BSIs in our hospitals still has room for improvement [6,7,52].

Notably, hub hospitals, despite treating more complex cases (including both SOT and hemopoietic stem cell—HSCT—recipients) and critically ill patients, achieved both lower in-hospital mortality rates (43% vs. 53%) and higher guidelines adherence. Consistent with the literature, these findings can partially be justified by the daily on-site presence of infectious disease (ID) specialists exclusively in the tertiary care center, while, in contrast, the peripheral hospitals depended exclusively on remote consultations during the study period [53,54,55,56].

To quantify adherence to guidelines recommendations, the EQUAL *Candida* score was developed. This tool assigns weighted points to each guideline principle according to the strength of the recommendation and its impact on outcomes, with a maximum of 22 points for patients with a CVC and 19 for those without [27].

Since its introduction into clinical practice, only a few studies with variegate backgrounds have evaluated EQUAL *Candida* score applicability and effectiveness with conflicting results.

A study from Portugal showed no statistically significant association between EQUAL *Candida* score and mortality [31], whereas Cuervo et al. documented that adherence to less than 50% of recommended practices significantly increased both early and overall mortality [32]. Similarly, a recent sub-analysis of the third ECMM *Candida* study found that each one-point decrease in the score was associated with a 9% higher mortality risk in patients with a CVC, and an 8% increase in those without endovascular devices at the moment of candidemia diagnosis [22].

A review of the literature revealed no other Italian studies evaluating the impact of the EQUAL *Candida* score on clinical outcomes. The only similar but not directly comparable study identified was the one performed by Vena and colleagues in 2020 in which the following implementation of an ID specialist-guided *Candida* BSI management bundle demonstrated a favorable effect on survival [33].

**Figure 2:**
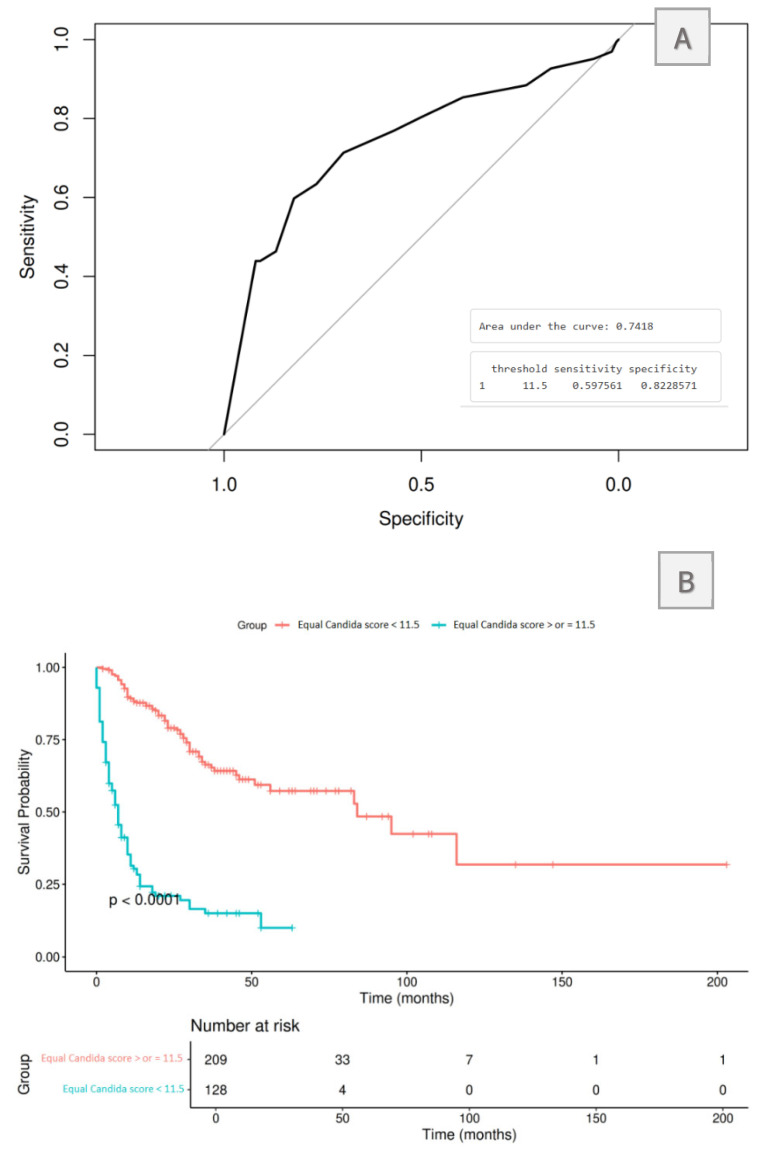
ROC curve and Kaplan–Meier analysis were performed using a cut-off of EQUAL Candida score of 11.5. (**A**). ROC curve analysis demonstrated an AUC of 0.741 after applying a cut-off of 11.5, leading to sensitivity and specificity levels of 60% and 82%, respectively. (**B**). Similarly, the Kaplan–Meier curves show statistically significant differences between mortality when applying this cut-off.

In our study, the EQUAL *Candida* score was predictive for in-hospital survivability, with higher scores associated with better short- and long-term survival outcomes. Statistically significant differences were observed particularly in patients not presenting with an endovascular device at the time of candidemia diagnosis. A logistic regression model was performed to assess the probability of in-hospital mortality based on the EQUAL *Candida* score. ROC curve and Youden index analysis identified an EQUAL *Candida* score <11.5 as the optimal mortality-predictive cut-off and multivariate Cox regression confirmed it as an independent risk factor for in-hospital mortality (HR 3.51, 95% CI: 2.34–5.27; *p* < 0.001), even after adjusting for potential confounders, including CVC presence and hospital level of care [Figure 2, Table 4 and Table 5].

These results are consistent with previous studies [23,24,57], where a proportional association between EQUAL *Candida* score and survival rates was observed with a varying cut-off threshold. A study from a Korean tertiary care center found that using a cut-off score of <15 in patients presenting with a CVC at the time of candidemia diagnosis was associated with significantly worse outcomes [24]. Similarly, Huang et al. observed comparable results, applying a cut-off score of 10 in a study conducted in the Taiwanese populations [23].

In our study, the administration of echinocandins as first-line antifungal agents resulted in a 2.6-fold higher probability of survival compared to when fluconazole was used, a result consistently observed across all three follow-up evaluations [azoles: intra-hospital mortality rate: 48.8% (N = 60/123), 30-day mortality: 60.16% (N = 74/123), 90-day mortality: 66.7% (N = 82/123) vs. echinocandin: intra-hospital mortality rate: 38.6% (N = 66/171), 30-day mortality was 36.25% (N = 62/171), 90-day mortality: 48.5% (N = 83/171)]. This aligns with evidence of the superior fungicidal activity of echinocandins in both neutropenic and non-neutropenic populations—even when treating azole susceptible *Candida* spp strains [7]. Nonetheless, fluconazole was the predominant empiric therapy in peripheral hospitals, diverging from ESCMID recommendations and potentially contributing to the poorer outcomes registered in secondary-care facilities.

On the other hand, echinocandin use has been associated with prolonged hospital stays [22], a concern likely exacerbated in our reality by low de-escalation rates—partly due to delays in confirming culture negativity (usually 5–7 days after the date of blood culture collection).

Notably, both ophthalmologic (OR = 12.48) and echocardiographic evaluations (OR = 6.79) were associated with improved clinical outcomes [Table 1]. Although already documented in other clinical studies [13], we believe these findings reflect broader adherence to EQUAL *Candida* score components rather than direct protective effects.

*Candida* endophthalmitis is a severe sight-threatening complication, and current guidelines recommend performing a dilated funduscopic examination within seven days after initiating an adequate antifungal therapy in non-neutropenic patients—delaying it until neutrophil recovery otherwise [25,26,51]. However, similar to our study, adherence to this practice remains globally low (30–50%), with no clear link to increased risk of ocular complications [6,7,22,23,24,31,57]. Recent meta-analyses report an overall incidence for *Candida* endophthalmitis of less than 1.8% of cases, often with presenting symptoms such as vision loss, photophobia, or floaters [58,59,60]. As a result, several experts have proposed the adoption of a symptom-guided, cost-effective approach—limiting ophthalmologic evaluations to non-neutropenic patients—capable of reporting ocular symptoms and provided ophthalmologists support is readily accessible to ensure prompt diagnosis and treatment initiation [7,33].

In our study, full 14-day therapy was associated with a four-fold increased survival benefit compared to shorter regimens, whereas extending treatment duration beyond 14 days offered no additional benefit [Table 1]. Similar to bacterial infections, limiting antifungal exposure is essential to reduce selective pressure, hence, the optimal duration of antifungal therapy has become a matter of debate, as shown by the emergence of recent studies supporting the use of shorter antifungal courses in non-neutropenic patients with adequate source control and uncomplicated disease [61,62]. Despite appearing to support the adoption of a more traditional therapeutic approach, our findings should be interpreted with caution since the exact duration of candidemia could not always be determined, as follow-up blood cultures were missing in 33% of cases [Table 2]. Therefore, we sustain the need for larger and potentially prospective case–control studies in the near future to establish the optimal duration of *Candida* BSIs infections.

Strengths and Limitations

Limitations of this study include its retrospective nature, the limited number of patients, a heterogeneous patient population, as well as varying follow-up times, and missing data. However, these limitations reflect the real-life context in which the data were collected, providing a more accurate representation of the local landscape.

Despite the fact that standard fluconazole doses are commonly used in our facilities, the exact doses prescribed were not systematically measured, limiting deficiencies in load-dosing administration assessment and possible underdosing evaluation.

## 5. Conclusions

*Candida*-related infections remain a major global health issue, with high incidence, mortality, and substantial economic burden. As a disease intrinsically tied to healthcare innovations and associated with numerous unavoidable risk factors, the prevalence of *Candida* BSIs is expected to rise steadily.

Data extrapolated from national and supranational surveillance programs provide only a rough picture of regional *Candida* spp. distribution and resistance patterns, emphasizing the urgency of establishing local infection prevention and control (IPC) programs to guide evidence-based management protocol development, ensure timely detection of shifts in local epidemiology, and prevent local outbreaks. Strict monitor of the adoption of best practices in *Candida* BSIs management should be encouraged to promptly identify potential areas of improvement in direct patients’ care and also to reinforce antifungal stewardship practices’ fulfillment.

In our region, the role of the ID specialists has proven to be fundamental in enhancing guideline compliance, and especially in promoting timely endovascular removal and appropriate empirical antifungal therapy prescription. Building on these findings, we have expanded on-site ID consultation services to spoke hospitals, alongside launching educational initiatives to train healthcare personnel in implementing best practices for *Candida* BSI management. We hope to demonstrate the beneficial impact of these measures in the near future.

To conclude, early diagnosis, strict adherence to clinical guidelines, optimization of treatment strategies according to local epidemiology, and rigorous implementation of antifungal stewardship within a One Health approach are essential measures to improve survival rates and reduce the global impact of the IC threat.

## Figures and Tables

**Table 1:** Univariate analysis of protective factors for 30-day mortality among patients diagnosed with *Candida* BSI between 2018 and 2022 (N = 341). Univariate logistic regression was performed with the outcome variable defined as “30 days survival”. Therefore, variables with an OR > 1 should be considered as protective factors, while those with an OR < 1 as risk factors.

Univariate Analysis			
Characteristics	OR	95% CI	*p*-Value
**Patients’ characteristics**			
Age	0.95	0.94–0.97	<0.0001
Female sex	0.66	0.43–1.01	0.061
CCI	0.82	0.76–0.89	<0.0001
**Risk factors**			
Central Venous Catheter (CVC)	0.89	0.57–1.42	0.650
ICU admission	2.56	1.58–4.20	<0.0001
Antibiotic treatment in the previous 30 days	2.15	0.93–5.41	0.0838
Extra–abdominal surgery	1.95	0.97–3.97	0.063
Metastatic solid tumor	0.49	0.25–0.94	0.037
Hematological malignancies	1.18	0.44–3.22	0.737
Solid organ transplant	1.04	0.19–5.70	0.959
Chronic renal impairment	0.96	0.52–1.74	0.882
Chronic hepatopathy	1.42	0.58–3.57	0.441
Diabetes mellitus	0.74	0.42–1.28	0.283
Chronic steroid treatment	0.83	0.20–3.19	0.783
High–dose steroid treatment	0.70	0.41–1.20	0.198
*Candida* colonization	1.41	0.76–2.62	0.278
Total parental nutrition (TPN)	0.76	0.49–1.19	0.235
**Hospital Units**			
Internal Medicine wards	0.36	0.23–0.56	0.0001
Oncology/Hematology	0.77	0.25–2.27	0.641
Other medical Units	1.69	0.86–3.43	0.134
Surgical Units	3.03	1.74–5.41	<0.0001
ICU	1.25	0.70–2.23	0.452
Hospitalization in hub hospital	2.08	1.35–3.22	<0.0001
**Microbiological characteristics**			
*Candida* species			
*C. albicans* infection	0.91	0.59–1.40	0.668
*C. parapsilosis* complex infection	2.49	1.39–4.59	0.003
*N. glabratus* infection	0.56	0.30–1.04	0.072
*C. tropicalis* infection	0.50	0.20–1.16	0.118
*P. kudriavzevii* infection	1.75	0.42–8.69	0.444
Biofilm production	1.17	0.66–2.09	0.591
***Candida* BSI management**			
First–line antifungal treatment			
Echinocandin	2.64	1.65–4.26	0.0001
Fluconazole	0.38	0.23–0.61	0.0001
Time from diagnosis and therapy initiation	0.58	0.36–0.93	0.0236
<=24 h from diagnosis	–	–	
24–72 h from diagnosis	1.45	0.91–2.32	0.116
>72 h from diagnosis	1.38	0.72–2.71	0.340
Antifungal treatment duration			
7–14 days	0.27	0.14–0.50	0.0001
14 days	4	1.62–12.12	0.006
>14 days	1.55	0.83–2.92	0.171
Timing of CVC removal			
<=24 h from diagnosis	1.47	0.76–2.85	0.248
24–72 h from diagnosis	1.50	0.76–2.99	0.244
>72 h from diagnosis	1.56	0.90–2.70	0.113
No removal	0.31	0.17–0.58	0.0003
Ophthalmoscopy	12.48	7.21–22.58	<0.0001
Echocardiography	6.79	4.07–11.71	<0.001

Legend: BSI = bloodstream infection, CI = confidence intervals, and OR = odds ratio.

**Table 2:** Guideline adherence rate in cohort study after excluding patients deceased within 7 days of diagnosis, stratified according to level of care of the admission hospital (hub vs. spoke). To minimize potential bias related to early mortality and ensure a valid comparison with similar studies [7,22,23,24,27,31,32,33], only patients who survived beyond seven days after candidemia diagnosis were included (N = 275). Only the factors written in black are included in the ECMM EQUAL *Candida* score. Factors written in grey were included to guarantee a better evaluation of statistically significant results. Legend: ^1^ n (%) and ^2^ false discovery rate correction for multiple testing.

	Guideline Adherence											
	All Hospitals				Hub Hospital				Spoke Hospitals			
	Overall N = 275 ^1^	Non-Survivors N = 98 ^1^	Survivors N = 177 ^1^	q-Value ^2^	Overall N = 156 ^1^	Non-Survivors N = 52 ^1^	Survivors N = 104 ^1^	q-Value ^2^	Overall N = 119 ^1^	Non-Survivors N = 46 ^1^	Survivors N = 73 ^1^	q-Value ^2^
**Microbiological recommendations**												
Initial blood culture	275 (100%)	98 (100%)	177 (100%)	1.000	156 (100%)	52 (100%)	104 (100%)	1.000	119 (100%)	46 (100%)	73 (100%)	1.000
Species identification	275 (100%)	98 (100%)	177 (100%)	1.000	156 (100%)	52 (100%)	104 (100%)	1.000	119 (100%)	46 (100%)	73 (100%)	1.000
Susceptibility testing	275 (100%)	98 (100%)	177 (100%)	1.000	156 (100%)	52 (100%)	104 (100%)	1.000	119 (100%)	46 (100%)	73 (100%)	1.000
**Medical treatment**												
First-line antifungal treatment	261/275 (95%)	92/98 (94%)	169/177 (95%)		149/156 (96%)	50/52 (96%)	99/104 (95%)		112/119 (94%)	42/46 (91%)	70/73 (96%)	
Echinocandin treatment	159/261 (61%)	53/92 (59%)	106/169 (63%)	0.6	117/149 (79%)	41/50 (82%)	76/99 (77%)	0.7	42/112 (38%)	12/42 (29%)	30/70 (43%)	0.3
Fluconazole treatment	102/261 (39%)	39/92 (42%)	63/169 (37%)	0.6	32/149 (21%)	9/50 (18%)	23/99 (23%)	0.7	70/112 (63%)	30/42 (71%)	40/70 (57%)	0.3
Treatment for 14 d after first negative follow-up culture	206/257 (81%)	52/92 (57%)	154/165 (93%)	<0.001	125/145 (86%)	30/47 (64%)	95/99 (96%)	<0.001	53/118 (45%)	30/46 (65%)	23/72 (32%)	<0.001
Step-down to fluconazole	46/159 (29%)	14/98 (26%)	32/177 (30%)		37/117 (32%)	12/41 (29%)	24/76 (32%)		9/42 (21%)	7/12 (58%)	2/30 (7%)	
**CVC management**												
Central Venous Catheter (CVC)	189/275 (69%)	69/98 (70%)	120/177 (68%)	0.8	104/156 (67%)	39/52 (75%)	65/104 (63%)	0.3	85/119 (71%)	30/46 (65%)	55/73 (75%)	0.4
CVC Removal (total)	152/189 (80%)	53/69 (77%)	99/120 (83%)	0.6	88/104 (85%)	32/52 (82%)	56/65 (86%)	0.7	64/85 (75%)	21/30 (70%)	43/55 (78%)	0.5
≤24 h from diagnosis	44/152 (29%)	15/69 (22%)	29/120 (24%)	0.7	27/104 (13%)	11/52 (28%)	16/65 (25%)	0.7	17/85 (20%)	4/30 (13%)	13/55 (24%)	0.4
24–72 h from diagnosis	47/152 (31%)	19/69 (28%)	28/120 (23%)	0.7	31/104 (30%)	11/52 (28%)	20/65 (31%)	0.7	16/85 (19%)	8/30 (27%)	8/55 (15%)	0.4
>72 h from diagnosis	61/152 (40%)	19/69 (28%)	42/120 (35%)	0.7	30/104 (29%)	10/52 (26%)	20/65 (31%)	0.7	31/85 (36%)	9/30 (30%)	22/55 (40%)	0.5
No removal	37/152 (24%)	16/69 (23%)	21/120 (18%)	0.7	16/104 (10%)	7/52 (18%)	9/65 (14%)	0.7	21/85 (25%)	9/30 (30%)	12/55 (22%)	0.5
**Follow-up procedure**												
Follow-up hemoculture	213/275 (77%)	63/98 (64%)	150/177 (85%)	<0.001	140/156 (90%)	42/52 (81%)	98/104 (94%)	0.031	73/119 (61%)	21/46 (46%)	52/73 (71%)	0.018
Echocardiography	120/275 (44%)	18/98 (18%)	102/177 (58%)	<0.001	87/156 (56%)	16/52 (31%)	71/104 (68%)	<0.001	33/119 (28%)	2/46 (4.3%)	31/73 (42%)	<0.001
Ophthalmoscopy	110/275 (40%)	19/98 (19%)	91/177 (51%)	<0.001	67/156 (43%)	10/52 (19%)	57/104 (55%)	<0.001	43/119 (36%)	9/46 (20%)	34/73 (47%)	0.012

**Table 3:** Patients’ characteristics, clinical, and microbiological outcomes and *Candida* BSI approaches in the three comparative groups. 2009–2011 data were retrieved from reference [34].

	Time Periods’			
	2009/2011	2018/2019	2020/2022	*p*-Value
	N = 99 ^1^	N = 131 ^1^	N = 210 ^1^	
**Patients’ characteristics**				
Mean age (SD)	63 ± 22	72 ± 16 °	73 ± 14 §	<0.001 °
Sex Male, n (%)	68 (68.7%)	82 (62.6%)	117 (55.7%)	0.071
CCI score, median [IQR, Q1–Q3]	Not determined ^#^	5 [4,8]	5 [4,8]	1.000
**Risk factors**				
Cardiovascular disease	57 (57.6%)	43 (32.8%) °	83 (39.5%) §	<0.001
Solid tumor	37 (37.4%)	42 (32.1%)	58 (27.6%)	0.1431
Metastatic solid tumor	25 (25.3%)	21 (16.0%)	23 (11.0%) §	0.005
Solid Organ Transplant	3 (3.0%)	1 (0.8%)	5 (2.4%)	0.3014
Hematological malignancies	5 (8.1%)	2 (1.5%)	15 (7.1%) §	0.047
Chronic hepatopathy	20 (20.2%)	9 (6.9%) °	12 (5.7%) §	<0.01
Diabetes mellitus	25 (25.3%)	22 (16.8%)	41 (19.5%)	0.1694
HIV Infection	2 (3.0%)	0 (0.0%)	0 (0.0%) §	0.005
Steroid treatment	21 (21.2%)	3 (2.3%) °	6 (2.9%) §	<0.001
ICU admission	19 (19.2%)	33 (25.2%)	65 (31.0%)	0.089
Surgery	35 (35.4%)	59 (45.0%)	78 (37.1%)	0.1604
Antibiotic treatment in the previous 30 days	83 (83.8%)	126 (96.2%) °	191 (91.0%)	0.006
Total Parenteral Nutrition (TPN)	58 (58.6%)	88 (67.2%)	132 (62.9%)	0.2750
*Candida* colonization	58 (58.6%)	26 (19.8%) °	22 (10.5%) § *	<0.001
Endovascular device (CVC, PICC, midline)	24 (24.2%)	93 (71.0%) °	142 (67.6%) §	<0.001
**Microbiological characteristics**				
*C. albicans* infection	65 (65.7%)	78 (59.5%)	122 (58.1%)	0.3208
*C. parapsilosis* complex infection	16 (16.2%)	20 (15.3%)	38 (18.1%)	0.5493
*N. glabratus* infection	11 (11.1%)	20 (15.3%)	31 (14.8%)	0.4340
*C. tropicalis* infection	5 (5.1%)	13 (9.9%)	11 (5.2%)	0.1271
***Candida* BSI management**				
Time from diagnosis and therapy initiation >48 h	40 (40.4%)	32 (28.1%) °	52 (28.7%) §	0.008
**Clinical outcome**				
In-hospital mortality rate	50 (50.5%)	63 (48.1%)	102 (48.6%)	0.6486
Medical Units mortality rate	43 (43.4%)	49 (37.4%)	76 (36.2%)	0.6597
Surgical Units mortality rate	3 (3.0%)	5 (3.8%) °	10 (4.8%) §	<0.001
ICU mortality rate	6 (6.1%)	9 (6.9%) °	16 (7.6%) §	0.002
30 days after candidemia diagnosis mortality rate	52 (52.5%)	69 (52.7%)	105 (50.0%)	0.6597

Legend: [IQR, Q1–Q3] [interquartile range, 1st interquartile—3rd interquartile]; ^1^ n (%) and °—*p* < 0.05 in Group 1 vs. Group 2; §—*p* < 0.05 in Group 1 vs. Group 3; *—*p* < 0.05 in Group 2 vs. Group 3. ^#^ CCI score was not determined for 2009–2011 group due to the absence of data regarding the presence and severity of renal impairment. Post hoc comparisons were performed with Bonferroni correction.

**Table 4:** EQUAL *Candida* score values stratified according to clinical outcome and level of care of the admission hospital. To minimize potential bias related to early mortality and ensure a valid comparison with similar studies, only patients who survived beyond seven days after candidemia diagnosis were included (N = 275). ^1^ Median (Q1, Q3) and ^2^ Wilcoxon rank sum test.

EQUAL *Candida* Score											
Overall, N = 275											
With CVC N = 189 ^1^						Without CVC N = 86 ^1^					
14.0 (12.0–17.0)						15.0 (11.0–17.0)					
Survivors N = 120 ^1^			Non-survivors N = 69 ^1^		*p*-value ^2^	Survivors N = 57 ^1^			Non-survivors N = 29 ^1^		*p*-Value ^2^
15.0 (12.0–17.0)			14.0 (11.0–17.0)		<0.001	16.0 (14.0–17.5)			11.0 (8.0–15.0)		<0.001
**Hub Hospital,** N = 156 ^1^						**Spoke Hospitals,** N = 119 ^1^					
With CVC N = 104 ^1^			Without CVC N = 52 ^1^			With CVC N = 85 ^1^			Without CVC N = 34 ^1^		
17.0 (14.0–19.0)			16.0 (13.0–18.0)			12.0 (10.0–15.0)			13.0 (8.0–16.0)		
Survivors N = 65 ^1^	Non-survivors N = 39 ^1^	*p*-value ^2^	Survivors N = 39 ^1^	Non-survivors N = 21 ^1^	*p*-value ^2^	Survivors N = 55 ^1^	Non-survivors N = 30 ^1^	*p*-value ^2^	Survivors N = 18 ^1^	Non-survivors N = 16 ^1^	*p*-value ^2^
17.0 (14.5–19.0)	16.0 (13.0–19.0)	0.7	17.0 (14.0–18.0)	13.0 (13.0–16.0)	0.029	13.0 (10.0–15.0)	11.5 (8.0–14.0)	0.11	14.5 (14.0–17.0)	8.0 (8.0–10.5)	<0.001

**Table 5:** Multivariate analysis used for performing logistic regression model to assess the probability of in-hospital mortality based on the EQUAL *Candida* score. Legend: HR = hazard ratio and CI = confidence interval. ^1^ Median (Q1, Q3).

	EQUAL *Candida* Score		CVC		Hospital Level of Care	
	≥11.5	<11.5	Not in Place at the Time of *Candida* BSI Diagnosis	In Place at the Time of *Candida* BSI Diagnosis	Hub Hospital	Spoke Hospitals
HR ^1^	–	3.51	–	1.05	–	1.03
95% CI ^1^	–	2.34–5.27	–	0.74–1.47	–	0.74–1.44
*p*–value	–	<0.001	–	0.8	–	0.8

## Data Availability

Data supporting reported results can be found in Università degli Studi di Udine archives.

## Supplementary Materials

The following supporting information can be downloaded at: https://www.mdpi.com/article/10.3390/jof11050400/s1, Table S1. Patients’ features and ward distribution among *Candida* species; Table S2. Susceptibility of *Candida* Bloodstream Isolates to Antifungal Drugs According to EUCAST breakpoints v 9.0 (2018–2019) v 10.0 (2020–2022); and Table S3. MIC50, MIC90, and susceptibility of *Candida* strains to antifungals.

## Abbreviations

The following abbreviations are used in this manuscript:

BSIs	Bloodstream infections
CCI	Charlson Comorbidity Index
CVC	Central venous catheter
ECMM	European Center of Medical Mycology
ESCMID	European Society of Clinical Microbiology and Infectious Diseases
EU	III ECMM Candida study results
EUCAST	European Committee on Antimicrobial Susceptibility Testing
FDR	False discovery rate
IC	Invasive candidiasis
ICU	Intensive Care Unit
ID	Infectious disease
IDSA	Infectious Diseases Society of America
IQR	Interquartile range
IMWs	Internal Medicine wards
HFR	Hospitals in the Friuli region
HH	Hub hospital
HR	Hazard Risk
HSCT	Hemopoietic Stem Cell Transplant
L–AmB	Liposomal amphotericin B
MALDI-TOF	Matrix-Assisted Laser Desorption/Ionization Time-of-Flight mass spectrometry technology
MDR	Multidrug-resistant
MIC	Minimum Inhibitory Concentration
NAC	Non-albicans Candida
OR	Odds ratio
PICC	Peripherally inserted central catheter
SH	Spoke hospitals
SOT	Solid organ transplant
TPN	Total parenteral nutrition
XDR	Extensively drug-resistant
