# Supplementary figures and images for: The Impact of Epidemiological Trends and Guideline Adherence on Candidemia-Associated Mortality: A 14-Year Study in Northeastern Italy
Source: J Fungi (Basel). 2025 May 21;11(5):400. doi: 10.3390/jof11050400 (PMC12113541; doi:10.3390/jof11050400)

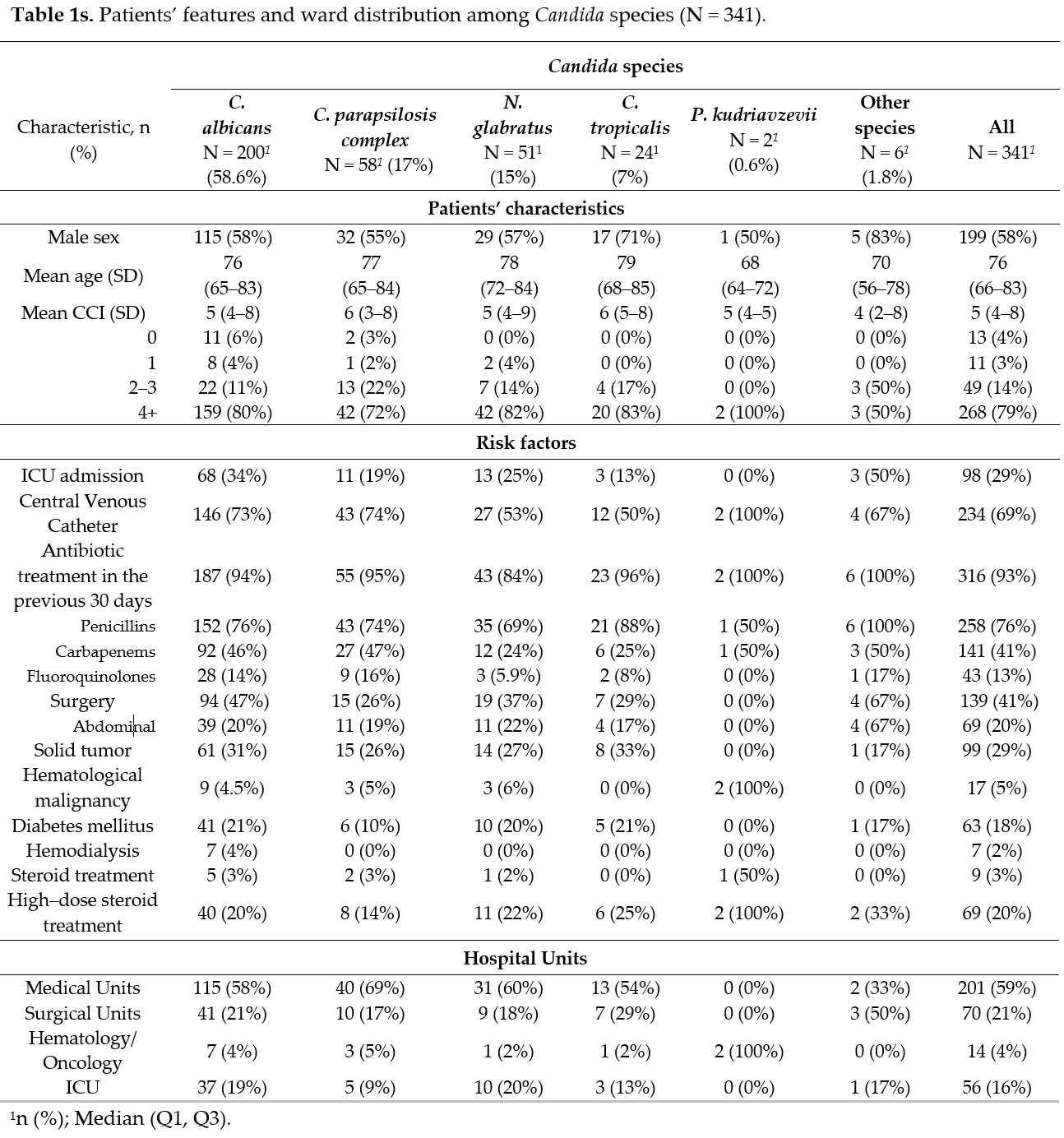

Supplement: Supplementary file 1 [file jof-11-00400-s001.zip › Table S1.png]

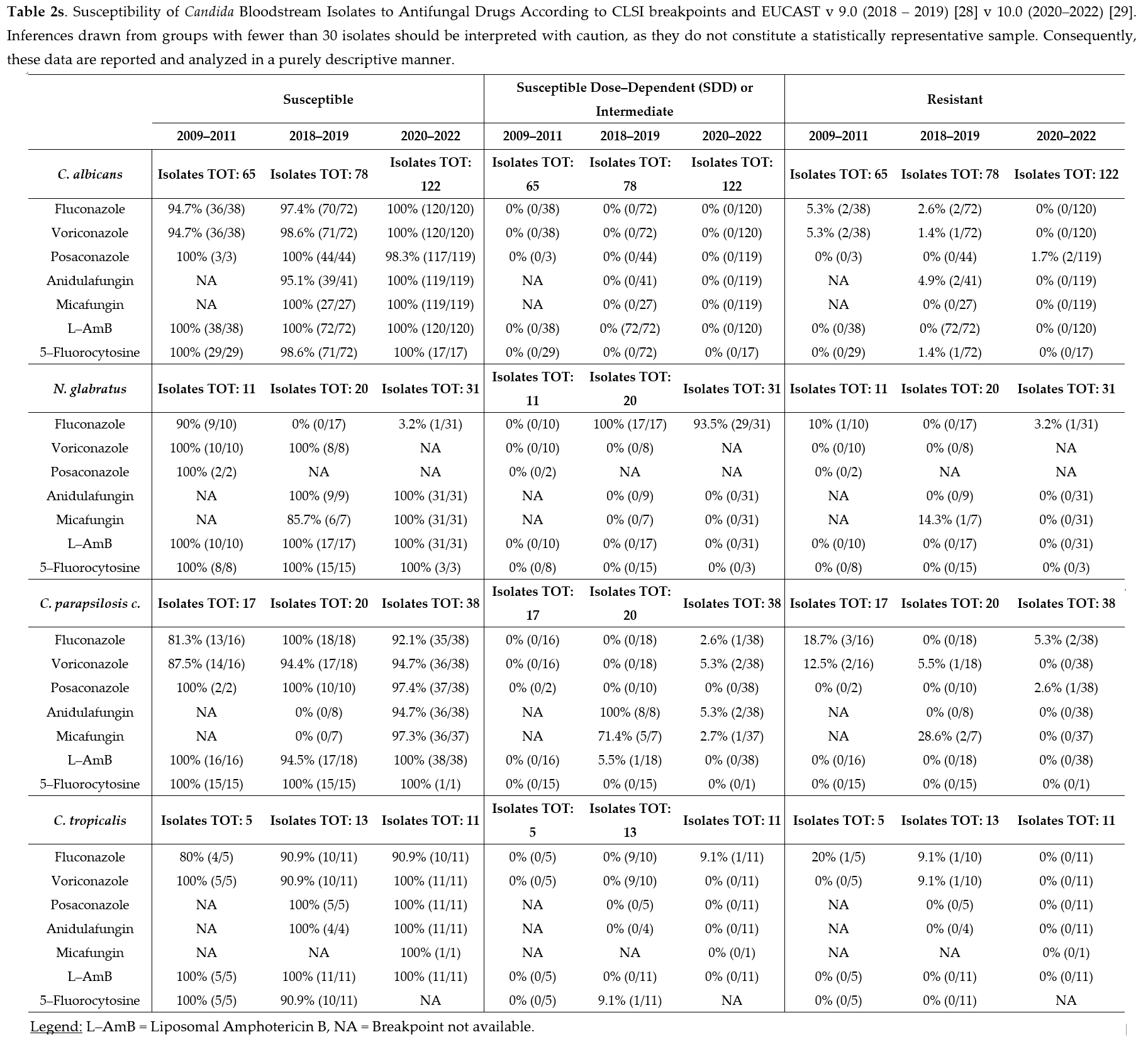

Supplement: Supplementary file 1 [file jof-11-00400-s001.zip › Table S2.png]

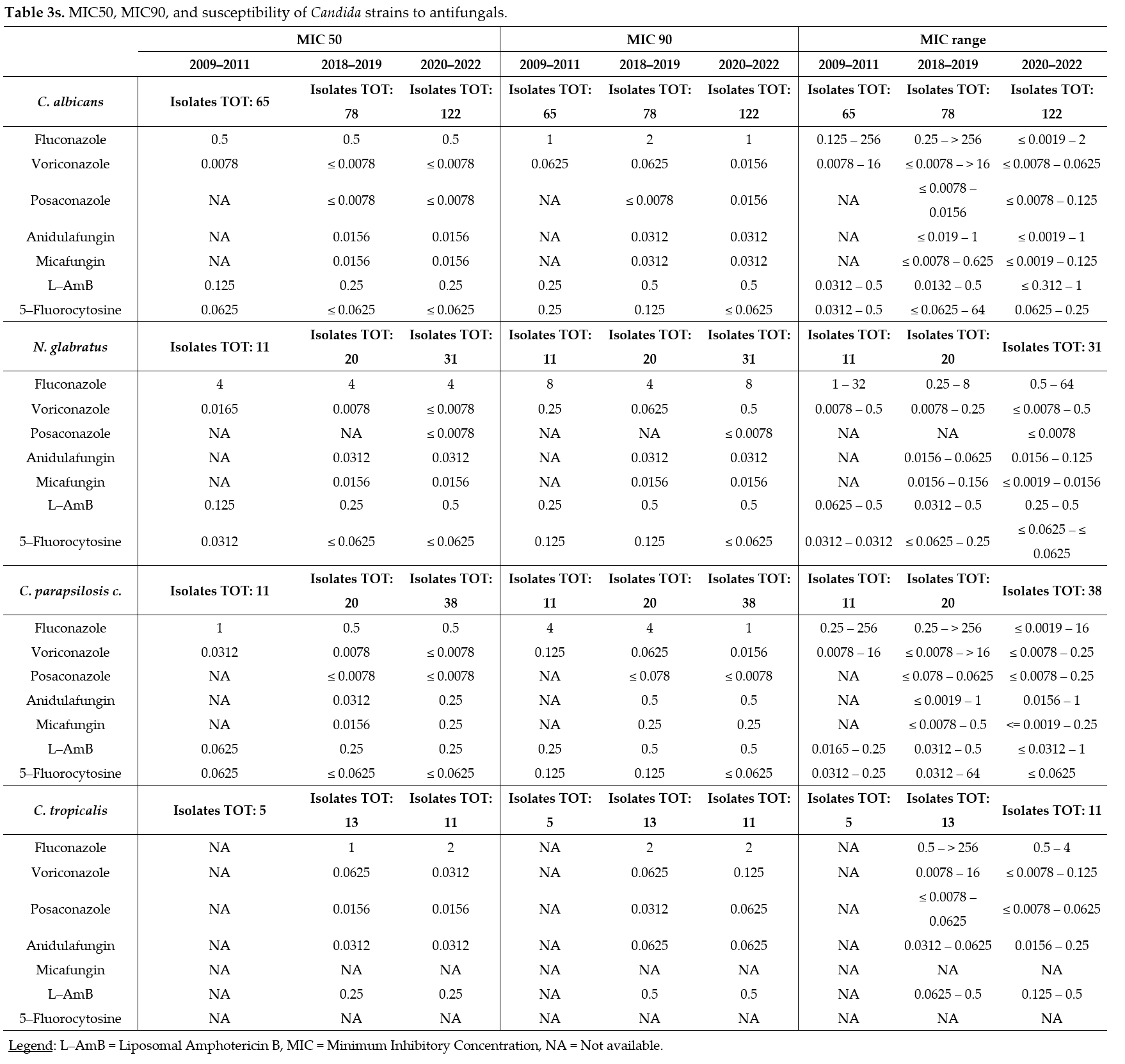

Supplement: Supplementary file 1 [file jof-11-00400-s001.zip › Table S3.png]
